# Brown Adipose Tissue Biodistribution and Correlations Particularities in Parathyroid Pathology Personalized Diagnosis

**DOI:** 10.3390/diagnostics12123182

**Published:** 2022-12-16

**Authors:** Wael Jalloul, Mihaela Moscalu, Irena Grierosu, Teodor Ionescu, Cati Raluca Stolniceanu, Mihai Gutu, Vlad Ghizdovat, Veronica Mocanu, Doina Azoicai, Radu Iliescu, Roxana Moscalu, Cipriana Stefanescu

**Affiliations:** 1 Department of Biophysics and Medical Physics-Nuclear Medicine, “Grigore T. Popa” University of Medicine and Pharmacy, 700115 Iasi, Romania; 2 Department of Preventive Medicine and Interdisciplinarity, “Grigore T. Popa” University of Medicine and Pharmacy, 700115 Iasi, Romania; 3 Department of Morpho-Functional Sciences (Pathophysiology), “Grigore T. Popa” University of Medicine and Pharmacy, 16, Universitatii Street, 700115 Iasi, Romania; 4 Department of Epidemiology, “Grigore T. Popa” University of Medicine and Pharmacy, 700115 Iasi, Romania; 5 Department of Pharmacology, “Grigore T. Popa” University of Medicine and Pharmacy, 700115 Iași, Romania; 6 Manchester Academic Health Science Centre, Cell Matrix Biology and Regenerative Medicine, The University of Manchester, Manchester M139PT, UK

**Keywords:** brown adipose tissue, obesity, hyperparathyroidism, parathyroid scan

## Abstract

Brown adipose tissue (BAT) participates in the regulation of whole-body metabolism by producing a variety of adipokines. This study investigates into the BAT pattern and the clinical aspects of overweight and obese (OOB) vs. non-obese (NO) hyperparathyroidism (HPT) patients with the aim of assessing the impact of BAT and obesity on HPT. Parathyroid scans performed on 441 HPT patients between 2015 and 2020 were retrospectively analyzed in order to select the images with active BAT. Based on their BMI, the patients with active BAT were divided into OOB vs. NO. The results showed that BAT was present in cervical and supraclavicular regions, with a single localization especially among NO vs. multiple sites among OOB. The (total counts/pixels)_BAT_/(total counts/pixels)_non-BAT_ ratio in the right cervical localization showed a significant difference between the groups with higher values in OOB. BMI, PTH, FT4, vitamin D, magnesium, creatinine, and urea had significant correlations with BAT ratios. The predictive values showed that right cervical ratios higher than 1.52 and right supraclavicular ratios lower than 1.15 indicated an increased probability of being OOB. The significant correlations between BAT activation in OOB vs. NO and HPT clinical parameters could be useful for developing potential treatments based on this tissue.

## 1. Introduction

HPT is a disorder that occurs when one or more parathyroid glands (PGs) overproduce parathyroid hormone (PTH) [1].

In 80% of cases, primary hyperparathyroidism (PHPT) is characterized by a single PG overgrowing, however, in 15–20% of cases there are several PG disorders [2].

In contrast with PHPT, the hormonal imbalances in secondary and tertiary HPT are brought about by an outside stimulus. One of the main causes of secondary HPT (SHPT) can be considered end-stage renal disease (ESRD), which has a worldwide prevalence of 0.1% [3,4]. It was reported that 12–54% of ESRD patients had HPT with PTH levels above 32 pmol/L [4].

PTH levels beyond the normal value (PTH > 55 pg/mL) are present in more than 80% of patients with a glomerular filtration rate (GFR) of less than 20 mL/min/1.73 m^2^ [4,5].

Obesity and PHPT appear to be related, albeit the exact mechanism of this association is still unknown [6]. This connection was first reported in studies looking into postmenopausal women with PHPT [6,7]. Subsequent research into the field unveiled that patients with severe obesity-related PHPT have bigger PGs and higher PTH levels. Some experts believe obesity to be directly linked with PHPT due to its significant prevalence in people with PHPT [6].

As the prevalence of obesity has reached epidemic/pandemic proportions worldwide, new strategies which aim to offer solutions against this disease have been considered, based, for example, on generating endogenous energy. Thus, BAT, which promotes weight loss by rising energy consumption, has the potential to be a key element in targeting obesity [8,9,10,11,12,13].

BAT is predominantly activated in infants and small hibernating mammals, representing up to 5% of body weight in neonates; however, it physiologically decreases by apoptosis [14] in adulthood with a possible persistence or reactivation in some adults [15,16].

By the metabolism of its abundant mitochondria, BAT generates heat and delivers it through vascularization to produce non-shivering thermogenesis, then participates in the body’s thermoregulation [15,17,18,19].

Current studies on genetic animal models demonstrate that, by producing a variety of adipokines, BAT also operates as an endocrine organ that participates in the regulation of the whole body’s metabolism. This tissue might contribute, for example, to glucose homeostasis and insulin sensitivity, representing a potential strategy to treat Type 2 Diabetes Mellitus [20,21,22,23].

**Figure 1:**
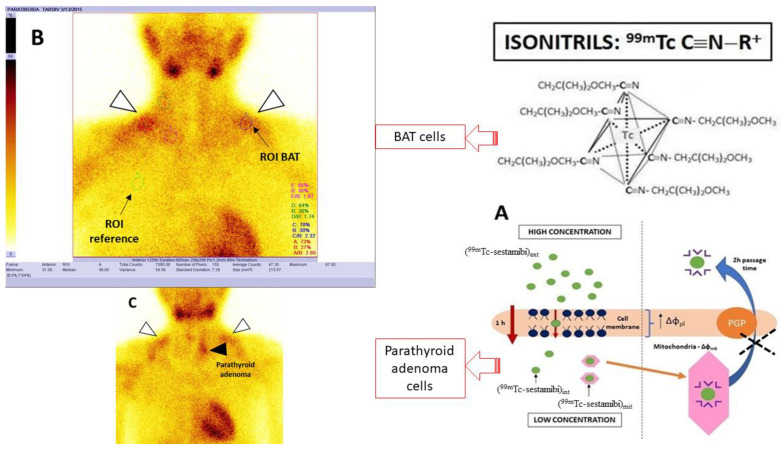
Schematic representation of ^99m^Tc-sestamibi uptake mechanism (**A**) which appears to be similar in active BAT and parathyroid adenoma (visible in cervical and supraclavicular localization in the PS (**B**) and cervical regions in (**C**)) and parathyroid adenoma (detected in PS (**C**)).

In non-invasively-localized preoperative/recurrent postoperative parathyroid adenomas in patients with HPT, dual-phase ^99m^Tc-isonitriles (either ^99m^Tc-sestamibi or ^99m^Tc-tetrofosmin) scintigraphy is frequently used [24,25,26]. ^99m^Tc-sestamibi crosses the cell membrane by a simple diffusion mechanism driven by the electrochemical gradient of the negatively charged molecule. It is then captured intracellularly in the mitochondria by a similar mechanism [27,28,29] (Figure 1).

The abundant vascularization and the high number of mitochondria could define an increased ^99m^Tc-sestamibi uptake in hyperfunctional parathyroid glands and adenomas. Moreover, it provides important information about active BAT blood flow and its energy metabolism [30,31].

To better understand how the correlation between BAT and body weight could interfere with the personalized diagnosis in HPT patients, we studied BAT patterns in a group of patients with parathyroid pathologies (PP) using non-invasive nuclear imaging. Taking into account that the spread of its activation among the population and its metabolic mechanisms are still not clarified [32], we also tried to elucidate potential correlations between BAT’s biodistribution and patients’ clinical parameters in order to reach a new possible personalized treatment approach based on the activation of this tissue.

## 2. Materials and Methods

- **Patients**

The study included 441 patients with various types of HPT referred by the Endocrinology Department of “St. Spiridon” County Emergency University Hospital to the Nuclear Medicine Laboratory, between 2015 and 2020, for dual-phase ^99m^Tc-sestamibi Parathyroid Scans (PS) to identify potential parathyroid adenomas or hyperfunctional PGs. The diagnosis of HPT was based on the clinical features in medical records as well as laboratory findings (PTH > 55 pg/mL). In patients with active BAT, standard clinical criteria and comorbidities such as renal pathologies, hypertension, diabetes, osteoporosis, and thyroid pathologies were listed in an Excel table together with biochemical and blood analysis findings, in particular PTH, TSH, FT4, calcium, vitamin D, phosphorus, magnesium, urea, and creatinine.

BMI was determined as the weight (in kg) divided by the height square (in m^2^). Patients were divided into two groups based on their BMI: group I, NO, with BMI less than 25 kg/m^2^; group II, OOB, with BMI greater than 25 kg/m^2^.

The patients remained in our laboratory at an ambient temperature (19–23 °C), for the necessary time to take the anamnesis, to prepare the radiotracer and for the substance to bind correctly to its target tissue. Therefore, the outside temperature did not have a real impact on BAT expression.

All the examination procedures followed the institutional guidelines. Our Nuclear Medicine Laboratory is part of a university hospital and, before every examination, the patient gives his informed consent for the possible use of their medical records for research purposes. Special ethical approval was not required since the study was retrospective and anonymous.

- **^99m^Tc-sestamibi Parathyroid Scanning Protocol**

A combined 2-days protocol was performed, with the ^99m^Tc-pertechnetate (^99m^TcO4^-^) thyroid scintigraphy on the first day, in addition to early (10 min) and delayed (2 h) ^99m^Tc-sestamibi parathyroid images on the second day.

Following the standard method, ^99m^Tc-sestamibi was properly labelled using a 10 min boiling period in a boiling water bath to attain an efficiency exceeding 90% [32]. Labelling efficiency was evaluated by the recommended radiochromatography (radio-TLC), and quality control checks were conducted in accordance with the manufacturer’s guidelines. Following the European Association of Nuclear Medicine (EANM) practice guidelines for parathyroid imaging, the patients received a mean IV dose of 505,79 MBq—^99m^Tc-sestamibi (dose interval: 296–666 MBq) [25].

Anterior planar dual-phase imaging was achieved with standard parameters (128 × 128 matrix, with a 20% window centered around the 140-keV photopeak, using a low-energy, high-resolution parallel collimator), early 10 min and delayed 2 h, using a Siemens e.cam nuclear gamma camera (Siemens Medical Systems). Scans of the neck and chest areas were accomplished in the supine position, with the neck extended. Single-photon emission computed tomography (SPECT) images were made when more fields of view were needed for precise localization.

- **Image processing and interpretation (Figure 1)**

After analyzing a total number of 986 scans (493 for both early and delayed scans some patients underwent more than one PS during this period), two nuclear medicine physicians reported the presence or absence of BAT in the nuchal, supraclavicular, and mediastinal regions by taking into consideration this tissue’s characteristic distribution and the main reported areas of physiological ^99m^Tc-sestamibi uptake (in salivary glands, thyroid, heart, gastrointestinal tract, and muscles). A third nuclear medicine physician was consulted to resolve a possible disagreement.

In the process of analyzing the included scans with active BAT, we studied this tissue’s pattern through measuring the total counts and pixels (given by the software of the Gamma Camera), by drawing a Region Of Interest (ROI) (mean value (mv) of 337.03 ± 67.23 mm^2^) in every BAT localization. Each ROI_BAT_ was reported to an equal ROI in a non-BAT reference area (right hemithorax) in which the presence of BAT has never been mentioned in the literature.

The (total counts/pixels)_BAT_/(total counts/pixels)_non-BAT_ ratio was used in order to identify potential correlations between BAT biodistribution and patients’ clinical parameters.

- **Statistical Analysis**

The statistical data analysis was performed using STATA 16 software (StataCorp LLC, 4905 Lakeway Drive, College Station, Texas 77845-4512, USA) and SPSS 26 (IBM Corporation, New Orchard Road Armonk, New York 10504-1722, USA). The continuous variables were presented as mean (deviation standard) or median (interquartile range) and the categorical variables were presented as numbers (frequencies). The comparison tests applied for the continuous numerical variables were selected based on the distribution of the series values and the number of cases included in the analysis. For the continuous numerical variables, the Wald-Wolfowitz Runs Test and Levene Test of Homogeneity of Variances were applied. The Kolmogorov–Smirnov test was applied to verify the normal distribution of the variables. The categorical variables were analyzed using the Pearson Chi-square test. The predictive power was evaluated based on the receiver operating characteristic (ROC) curve, taking into account the area under the curve (AUC). *p*-values of less than 0.05 were considered for statistical significance.

## 3. Results

The accumulation of ^99m^Tc-sestamibi in active BAT mitochondria was visualized in 56 delayed scans (5.68% of total images, 11.36% of delayed scans) of 56 patients with a mean age of 53.18 years (group I: NO; 48.3 ± 18.3 years vs. group II: OOB; 58.4 ± 9.3 years). The demographic/clinical characteristics of these patients are listed in Table 1.

We noticed the predominance of females (85.7%), with a greater percentage in group II (96.3% vs. 79.3% in group I). The BMI mean value (mv) was 25.3 ± 4.9 kg/m^2^ with 21.7 ± 2.6 kg/m^2^ in group I vs. 29.1 ± 3.67 kg/m^2^ in group II. HPT was recorded in 87.5% of subjects (69.6% primary), the rest of the cases presenting parathyroid adenomas, without significant differences between the two groups. Comorbidities including diabetes (10.7% of cases), renal pathologies (like chronic kidney disease), hypertension, and osteoporosis (80.4% of cases) incidence were similar amongst the groups. Endocrine comorbidities showed no distinction between the two groups, 60.7% of patients presented thyroid pathologies, mainly nodular goiter (39.28% of cases) and Hashimoto’s disease (10.7%). Apart from PTH (higher in group I with mv = 719.5 ± 1034.3 pg/mL vs. 174.5 ± 128.9 pg/mL in group II, *p* = 0.0314; more than 25% of NO patients had values higher than 1290 pg/mL) (Table 1) and creatinine (greater in group I with mv = 2.34 ± 3.06 mg/dL vs. 0.89 ± 0.31 mg/dL in group II, *p* = 0.0354), all other biochemical and blood analysis findings were not significantly different amongst BMI groups.

Following the PS analysis (Table 2), we noticed the presence of parathyroid adenoma(s) in 82.1% of images. BAT had symmetric distribution in 92.9% and homogeneous in 42.9%. This tissue was recorded in cervical and supraclavicular regions with a single localization in 73.2% of scans (87.8% cervical), and a high frequency among NO patients (*p* = 0.0211), whereas all the rest of the images presented multiple locations, with a preponderance among OOB cases (*p* = 0.0228). The highest (total counts/pixels)_BAT_/(total counts/pixels)_non-BAT_ ratio was identified in the supraclavicular region with 2.59 vs. 2.49 in the cervical area. The ratio for the right cervical localization showed a significant difference between the groups, with a higher value in group II (1.53 ± 0.23 vs. 1.45 ± 0.31, *p* = 0.0314).

**Figure 2:**
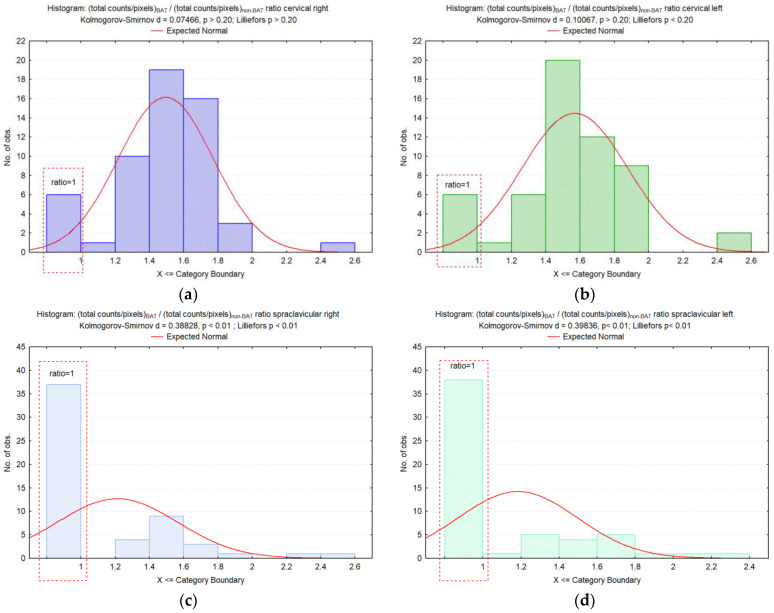
Histograms for the values of (total counts/pixels)_BAT_/(total counts/pixels)_non-BAT_ ratio in the cervical (**a**,**b**) and supraclavicular localizations (**c**,**d**). (*A ratio of 1 is obtained when BAT is not activated in the concerned localisation*: (total counts/pixels)_BAT_ = (total counts/pixels)_non-BAT_).

Concerning the values’ distribution of (total counts/pixels)_BAT_/(total counts/pixels)_non-BAT_ ratio, the cervical ratios (right and left) showed a high frequency of cases with values between 1.4 and 1.8. A significant number of cases had values ranging between 1.4 and 1.6 for the right supraclavicular ratio, while the left supraclavicular ratio had values distributed between 1.2 and 2.4 (Figure 2).

**Figure 3:**
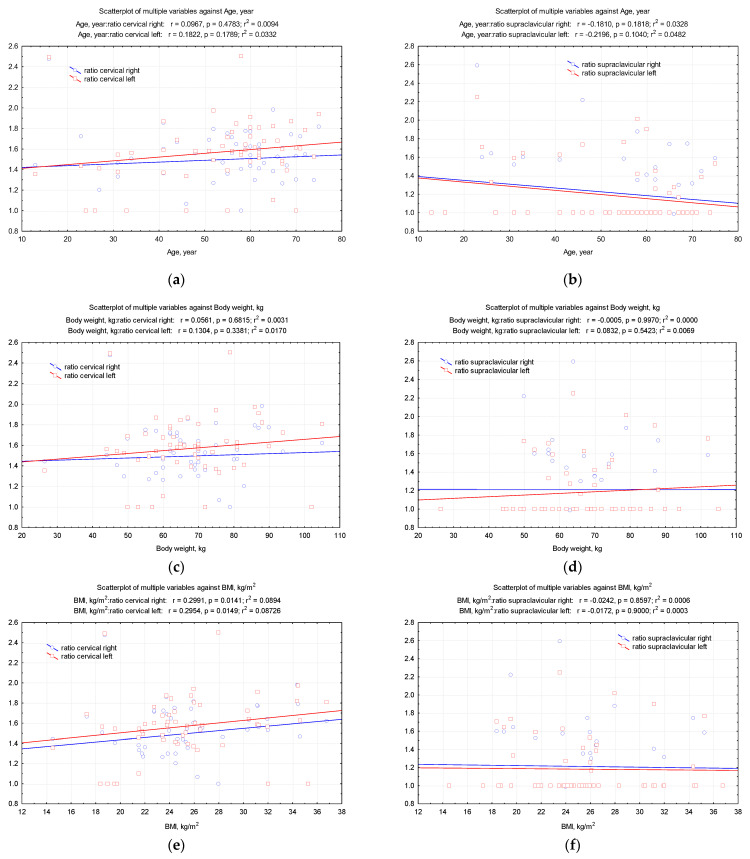
Pearson correlations between the demographic aspects (**a**,**b**) age, (**c**,**d**) body weight, (**e,f**) BMI and the values of (total counts/pixels)_BAT_/(total counts/pixels)_non-BAT_ ratio.

The correlations between this BAT ratio and the demographic aspects showed a significant relation between the right and left cervical BAT ratios and the BMI values (the increase in BMI was followed by the increase in right and left cervical BAT ratios; r = −0.299, *p* = 0.014 on the right; r = −0.295, *p* = 0.014 on the left) (Figure 3).

**Figure 4:**
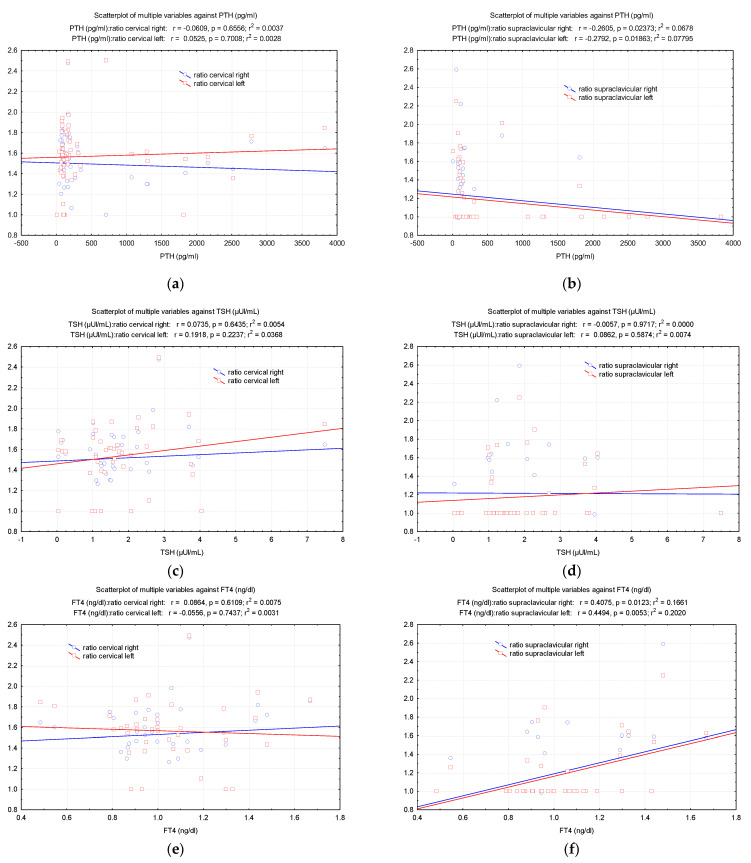
Pearson correlations between some endocrine hormones (**a**,**b**) PTH, (**c,d**) TSH, (**e,f**) FT4 and the values of (total counts/pixels)_BAT_/(total counts/pixels)_non-BAT_ ratio.

It was shown that an increase in PTH correlates with a decrease in the right and left supraclavicular BAT ratios (r = −0.260, *p* = 0.023 on the right; r = −0.279, *p* = 0.018 on the left), however, these ratios rise with an increase in FT4 (r = 0.407, *p* = 0.012 on the right; r = 0.449, *p* = 0.005 on the left) (Figure 4).

**Figure 5:**
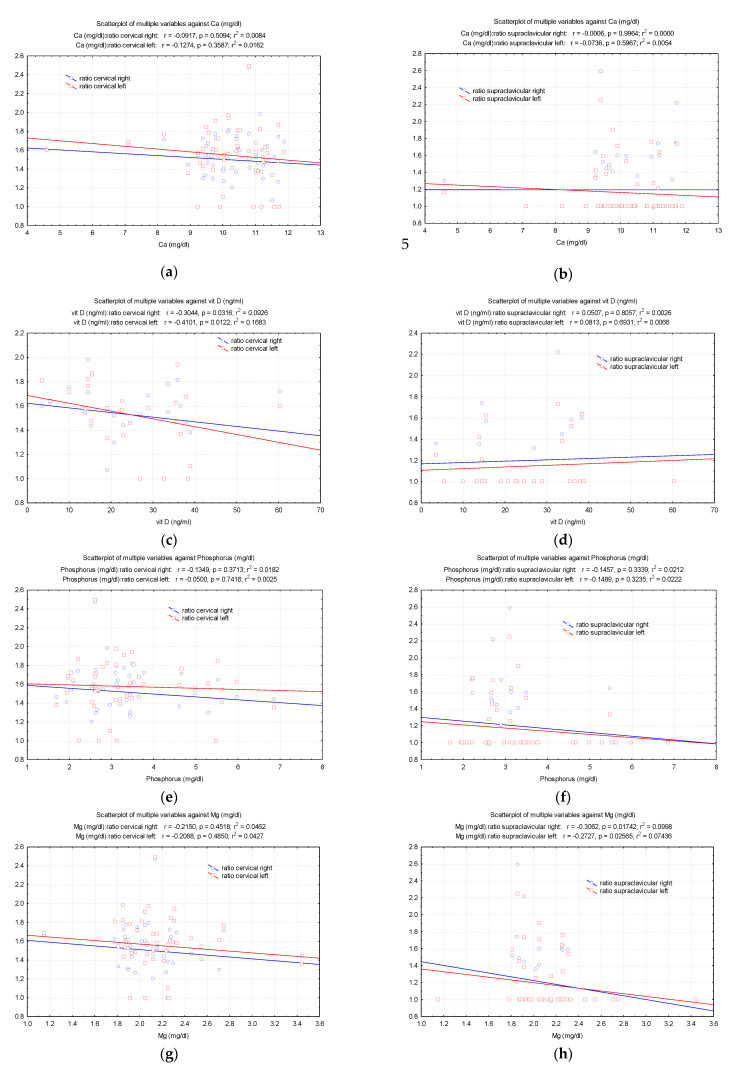

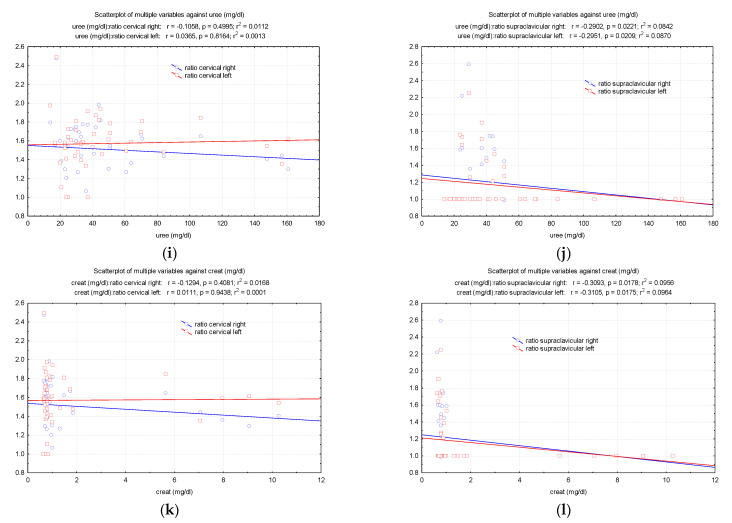
Pearson correlations between the biochemical/blood analysis (**a**,**b**) Ca, (**c**,**d**) vit D, (**e**,**f**) Phosphorus, (**g**,**h**) Mg, (**i**,**j**) uree, (**k**,**l**) creat and the values of (total counts/pixels)_BAT_/(total counts/pixels)_non-BAT_ ratio.

As vitamin D increases, the right and left cervical BAT ratios decrease (r = −0.304, *p* = 0.031 on the right; r = −0.410, *p* = 0.012 on the left). Furthermore, the increase in magnesium leads to a decrease in the right and left supraclavicular BAT ratios. It was noted that the increase in creatinine and urea was followed by a significant decrease in the right and left supraclavicular BAT ratios (Figure 5).

**Figure 6:**
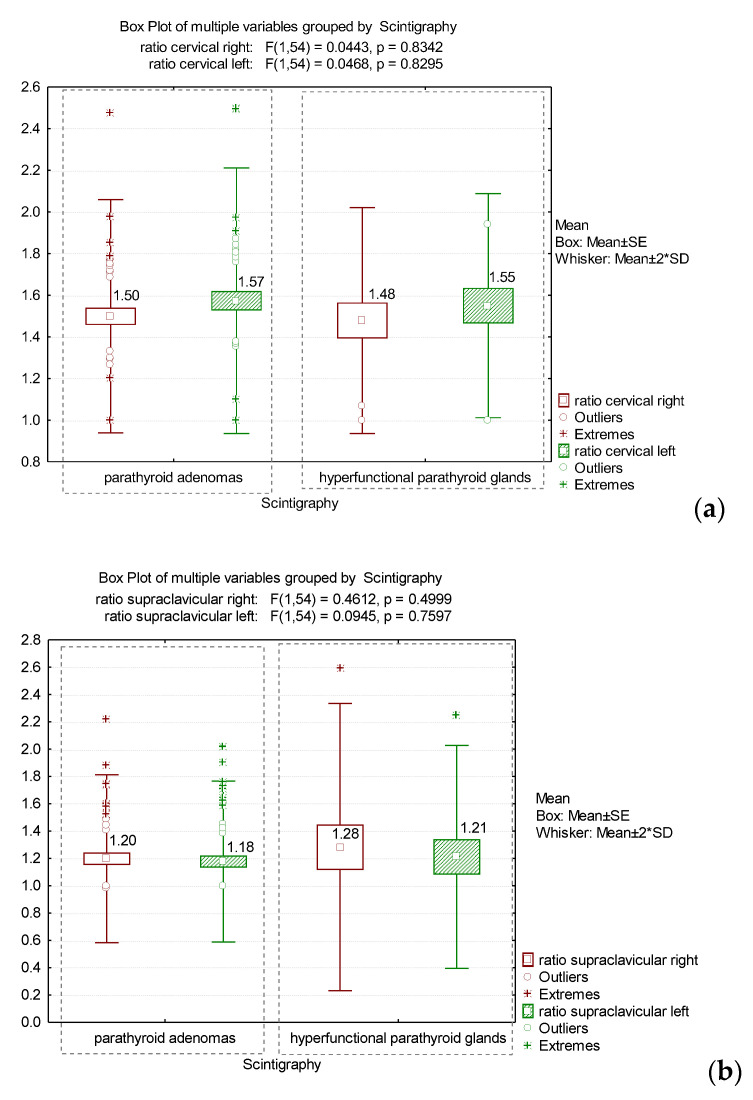
The comparison between mean values of the (total counts/pixels)_BAT_/(total counts/pixels)_non-BAT_ ratio in the cervical (**a**) and supraclavicular regions (**b**), and the scintigraphic diagnosis.

The values of (total counts/pixels)_BAT_/(total counts/pixels)_non-BAT_ ratio in the right and left cervical localization, in addition to the right and left supraclavicular regions, did not show significant differences with the scintigraphic diagnosis (parathyroid adenomas and hyperfunctional PGs) (*p* > 0.05) (Figure 6).

**Figure 7:**
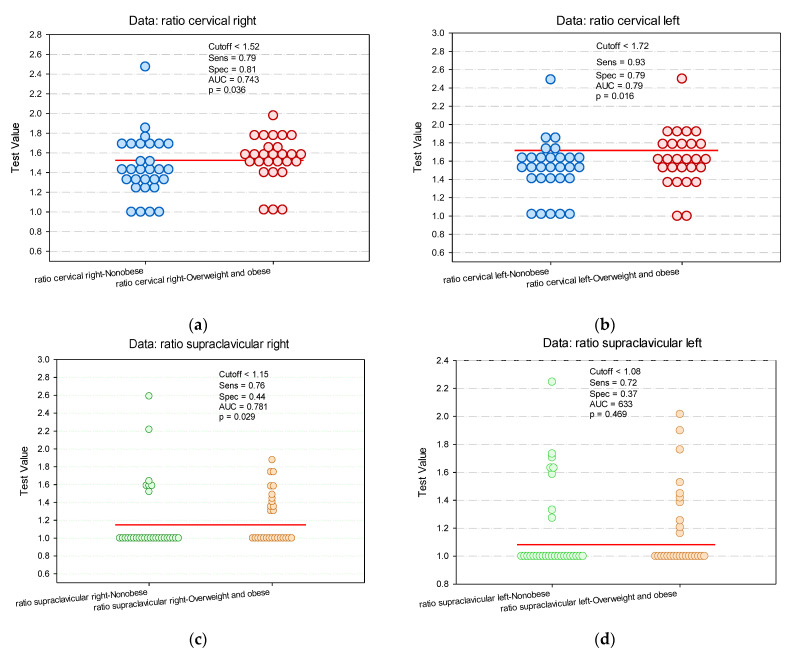
Paired histograms for estimating the cut-off value of (total counts/pixels)_BAT_/(total counts/pixels)_non-BAT_ ratio in cervical (**a**,**b**) and supraclavicular localizations (**c**,**d**).

The analysis of the predictive values of the (total counts/pixels)_BAT_/(total counts/pixels)_non-BAT_ ratio (Figure 7) indicated a cutoff for the right cervical ratio of 1.52 (AUC = 0.74, *p* = 0.036), with a sensitivity (Se) of 79% and a specificity (Sp) of 81%. Ratios higher than 1.52 indicated an increased probability of obesity. For the left cervical ratio, a cutoff of 1.72 was calculated with a Se = 93% and Sp = 79% (*p* = 0.016). In the supraclavicular localization, only the right supraclavicular ratio values presented a cutoff with significant predictive power (*p* = 0.029). Values lower than 1.15 indicated an increased probability of obesity (Se = 76%, Sp = 44%).

## 4. Discussion

Given the variations in its developmental, anatomical, and functional characteristics, adipose tissue is typically categorized as either white or brown [33].

White adipose tissue (WAT) accumulates triglyceride molecules as a source of energy, which it aims to deliver into the bloodstream through free fatty acids as a response to the lack of glucose provision [34]. Obesity and insulin resistance could be a direct result of an excess of this type of energy supply [35].

It is likely that obesity, through vitamin D deficiency and expansion of the parathyroid glands, contributes to PHPT [6,7]. In contrast, it has been proposed that PHPT may promote obesity. Adam MA et al. [6] demonstrated in their study that, regardless of vitamin D levels, obesity may impact parathyroid tumor (PT) growth. Larger PT weight, higher pre- and postoperative PTH, and more severe symptoms are all signs that severely obese patients (BMI ≥ 35 kg/m^2^) have a more severe disease pattern [6,7,36].

While WAT is responsible for storing and releasing lipids, BAT oxidizes lipids to produce heat. In addition to its ability to generate heat with the non-shivering process of thermoregulation, BAT contributes to the modulation of energy balance and insulin resistance. It also regulates the entire body’s metabolism by producing a variety of adipokines [19,20,21,22]. It has been demonstrated that this tissue’s activity has a more striking impact in lean subjects in comparison with obese ones [37,38,39,40], and an activated BAT by cold exposure expends up to several hundred kcal/day [41], representing the organ with the most important glucose/gram consumption, in this situation [42].

This tissue is characterized by multilocular adipocytes with large numbers of mitochondria (which give its specific name and color), expanded blood supply, and plentiful sympathetic noradrenergic innervations, such as b3-adrenergic receptors [43].

Unfortunately, the BAT physiological response to stimulation and its regulating mechanisms are still not elucidated [15,44]. It was shown in our work that the increase in BMI was followed by an increase in right and left cervical BAT ratios. Thus, these features spiked the pharmaceutical industry’s interest in developing pharmacologic agents that can activate and expand this tissue, for example through sympathetic stimulation, and integrate it into antiobesity and metabolic dysregulation therapeutical strategies [15,45,46,47]. Recent studies using ^18^F-fluorodeoxyglucose (^18^F-FDG) positron emission tomography/computed tomography (PET/CT) on healthy subjects proved that mirabegron, a β3-adrenergic receptor agonist, rises BAT activity and could be a promising agent for a potential treatment against metabolic diseases [48]. It was also mentioned that adenosine could have therapeutic implications by activating this type of fat and recruiting beige adipocytes through A2A receptors [49].

Due to the high concentration of mitochondria in active BAT, ^99m^Tc-sestamibi represents a suitable radiotracer for this tissue’s function detection. 

This agent is a lipophilic cationic radiotracer that passes the cellular membrane by passive transport to be captured into the mitochondria [27,50]. ^99m^Tc-sestamibi is able to visualize hyperfunctional PGs (with normal or ectopic localizations) and parathyroid adenomas due to their oxyphil cells that are overloaded in mitochondria, contrary to the normal PGs that have no uptake [50,51].

Knowledge about the BAT response to pharmacological stimulations is still very limited, thus the accessible ^99m^Tc-sestamibi scintigraphy could have a principal role in providing more information about its pattern and function.

The inducible “browning” of WAT is a phenomenon that is obtained after the stimulation, by a particular type of factor like cold exposure, of a certain population of WAT adipocytes in the presence of mitochondrial uncoupling protein 1 (UCP1). These adipocytes turn into a specific type of cell called “brite fat” or “beige fat”, which are different from the classic BAT from a developmental point of view [19,52,53,54].

PTH is a traditional calcium-regulating hormone whose primary effects on the kidney and bones has long been known [55]. Adipose tissue is another organ that PTH targets, according to research presented by He Y et al. [56]. In addition to the thyroid and catecholamine hormones, which are known to promote WAT browning/ BAT activation, PTH also facilitates these effects [48,56].

He Y et al. [56] demonstrated that in PHPT mice and patients the increased serum PTH levels stimulated the browning of adipose tissue, which resulted in higher energy expenditure, lower fat content, and, ultimately, lower body weight. The fact that in our study PTH had higher values in NO supports this hypothesis. However, many researchers revealed that serum PTH levels were positively correlated with body weight and body fat mass in people, contradicting the hypothesis of PTH browning effects [57,58]. Furthermore, Mendoza-Zubieta V et al. [59] showed that compared to healthy control subjects, PHPT patients had greater body weight, higher levels of WAT, and elevated prevalence of insulin resistance and metabolic syndrome. It is yet to be determined how increased PTH secretion contributes to the regulation of body weight in PHPT and how it affects the WAT browning/BAT activation.

The new pathogenic PTH effects on adipose tissue, previously described, led us to carefully re-evaluate the pathophysiological alterations in HPT, which is typically defined by hypercalcemia and its associated gastrointestinal, renal, and skeletal symptoms [55].

Differences in the metabolic indices between asymptomatic and symptomatic HPT patients were identified in various studies: It was demonstrated that asymptomatic PHPT patients had a higher prevalence of metabolic syndrome than symptomatic ones [60,61].

Therefore, the disease severity or stage/duration of its evolution could explain the opposition between the elevated body weight or the metabolic syndrome prevalence, described in PHPT by some researchers, and the body weight loss due to browning WAT/ BAT activation [36].

Crucially, the findings might serve as a reminder to endocrinologists to take into account the increased energy-consuming condition when treating patients with symptomatic PHPT, particularly in those with severe disease [56].

When evaluating the results of the current study, it is important to consider a number of limitations. Patients with PHPT are thought to lose weight as a result of hypercalcemia and its resulting gastrointestinal symptoms, such as nausea and vomiting [56]. Another thing that should be emphasized is the connection between thyroid diseases and weight loss in HPT. The small number of patients considered in the groups is explained by the fact that, in order to study the BAT pattern, we needed to include only patients with activated BAT. These patients represent a minority as it is already known that BAT could be activated in adults only in very specific situations. Given the small number of cases in the analyzed patient groups, statistical tests specific to this particularity (small samples) were applied to validate the hypotheses. Thus, in the univariate statistical analysis used for comparisons, the statistical power of the estimates was maintained at an acceptable level.

## 5. Conclusions

This research supports the hypothesis that there is a correlation between the pattern of BAT distribution in NO and OOB patients and the pattern of HPT. Furthermore, this type of fat can represent an important factor in the evolution of HPT. HPT severity or stage/duration of its evolution may have an impact, by activating BAT, on the patient’s weight status, implicitly on the treatment of obesity. Due to this association, BAT activation represents a candidate for a potential prospective therapeutic method/algorithm for obesity, in the context of certain types of parathyroid pathologies.

## Figures and Tables

**Table 1:** The demographic/clinical characteristics of patients with active BAT.

Clinical Characteristics	Total Patients *n* = 56	Nonobese Patients (BMI < 25 kg/m^2^) *n* = 29	Overweight and Obese Patients (BMI > 25 kg/m^2^) *n* = 27	*p*-Value
Age, median (IQR), year	58 (45–64)	52 (33–65)	60 (55–62)	0.062 *
Age, mean (SD), year	58.4 (15.4)	48.3 (18.3)	58.4 (9.3)	
Gender, female/male, *n* (%)	49/7 (87.5/12.5)	23/6 (79.3/20.7)	26/1 (96.3/3.7)	0.043 ^^^
Season, *n* (%)				
spring	19 (33.9)	15 (51.7)	4 (14.8)	0.014 ^^^
summer	14 (25)	5 (17.2)	9 (33.3)	
autumn	12 (21.4)	3 (10.4)	9 (33.3)	
winter	11 (19.6)	6 (20.7)	5 (18.6)	
Scintigraphy, *n* (%)				
parathyroid adenomas	46 (82.1)	23 (79.3)	23 (85.2)	0.564 ^^^
hyperfunctional parathyroid glands	10 (17.9)	6 (20.7)	4 (14.8)	
Body weight, median (IQR), kg	67.5 (58–75.5)	60 (53–64)	75 (70–86)	<0.001 *
Body weight, mean (SD), kg	67.5 (14.7)	58.1 (10.5)	77.8 (11.2)	
BMI, median (IQR), kg/m^2^	24.6 (21.9–27.3)	22.1 (19.6–23.8)	27.9 (25.9–31.9)	<0.001 *
BMI, mean (SD), kg/m^2^	25.3 (4.9)	21.7 (2.6)	29.1 (3.67)	
Comorbidities, *n* (%)	55 (98.2)	28 (96.5)	27 (100)	0.330 ^^^
Diabetes	6 (10.7)	2 (6.9)	4 (14.8)	0.335 ^^^
Renal pathologies	17 (30.4)	9 (31.1)	8 (29.6)	0.909 ^^^
Hypertension	19 (33.9)	8 (27.6)	11 (40.7)	0.298 ^^^
Osteoporosis	28 (50)	13 (44.8)	15 (55.6)	0.421 ^^^
PTH, mean (SD), pg/mL	456.7 (792.4)	719.5 (1034.3)	174.5 (128.9)	0.031 *
TSH, mean (SD), µUI/mL	1.86 (1.37)	2.03 (1.62)	1.62 (0.94)	0.254 *
FT4, mean (SD), ng/dL	1.04 (0.24)	1.05 (0.27)	1.03 (0.21)	0.524 *
Ca, mean (SD), mg/dL	10.21 (1.23)	10.01 (1.05)	10.42 (1.37)	0.275 *
Vitamin D, mean (SD), ng/mL	24.68 (12.84)	28.79 (13.94)	21.15 (11.12)	0.975 *
Phosphorus, mean (SD), mg/dL	3.35 (1.18)	3.73 (1.25)	2.87 (0.91)	0.625 *
Magnesium, mean (SD), mg/dL	2.13 (0.33)	2.19 (0.39)	2.04 (0.18)	0.360 *
Urea, mean (SD), mg/dL	46.65 (35.54)	54.62 (43.71)	36.57 (17.71)	0.098 *
Creatinine, mean (SD), mg/dL	1.70 (2.39)	2.34 (3.06)	0.89 (0.31)	0.035 *
Thyroid pathology, *n* (%)				
Hashimoto’s autoimmune thyroiditis	6 (10.7)	3 (10.3)	3 (11.1)	0.926 ^^^
Basedow’s disease	2 (3.6)	1 (3.5)	1 (3.7)	0.958 ^^^
Nodular goiter	22 (39.3)	10 (34.5)	12 (44.4)	0.445 ^^^

* Wald-Wolfowitz Runs Test, Levene Test of Homogeneity of Variances (*p* < 0.05). ^ Pearson Chi-square.

**Table 2:** BAT pattern and quantification.

	Total Patients	Nonobese Patients (BMI < 25 kg/m^2^) *n* = 29	Overweight and Obese Patients (BMI > 25 kg/m^2^) *n* = 27	*p*-Value
BAT localisation, *n* (%)				
Unique location	41 (73.2)	25 (86.2)	16 (59.3)	0.021 ^^^
Multiple locations	15 (26.8)	4 (13.8)	11 (40.7)	
BAT,				
homogeneous	24 (42.9)	10 (34.5)	14 (51.9)	0.189 ^^^
non-homogeneous	32 (57.1)	19 (65.5)	13 (48.1)	
BAT				
symmetric	52 (92.9)	28 (96.6)	24 (88.9)	0.265 ^^^
asymmetric	4 (7.1)	1 (3.4)	3 (11.1)	
(total counts/pixels)_BAT_/				
(total counts/pixels)_non-BAT_				
ratio **cervical right**				
median (IQR)	1.52 (1.34–1.65)	1.44 (1.29–1.66)	1.55 (1.46–1.63)	0.031 *
mean (SD)	1.49 (0.27)	1.45 (0.31)	1.53 (0.23)	
(total counts/pixels)_BAT_/				
(total counts/pixels)_non-BAT_				
ratio **cervical left**				
median (IQR)	1.58 (1.42–1.71)	1.54 (1.41–1.68)	1.59 (1.47–1.81)	0.412 *
mean (SD)	1.57 (0.31)	1.51 (0.31)	1.62 (0.30)	
(total counts/pixels)_BAT_/				
(total counts/pixels)_non-BAT_				
ratio **supraclavicular right**				
median (IQR)	1 (1–1.42)	1 (1–1.12)	1 (1–1.45)	0.284 *
mean (SD)	1.21 (0.35)	1.9 (0.41)	1.23 (0.29)	
(total counts/pixels)_BAT_/				
(total counts/pixels)_non-BAT_				
ratio **supraclavicular left**				
median (IQR)	1 (1–1.30)	1 (1–1.27)	1 (1–1.38)	0.992 *
mean (SD)	1.18 (0.31)	1.18 (0.38)	1.19 (0.31)	

* Wald-Wolfowitz Runs Test, Levene Test of Homogeneity of Variances (*p* < 0.05). ^ Pearson Chi-square.

## Data Availability

The data presented in this study are available on request from the corresponding author.

## References

[1] Messa P., Alfieri C.M. (2019). Secondary and Tertiary Hyperparathyroidism. Front. Horm. Res..

[2] Bilezikian J.P., Bandeira L., Khan A., Cusano N.E. (2018). Hyperparathyroidism. Lancet.

[3] Hill N.R., Fatoba S.T., Oke J.L., Hirst J.A., O’Callaghan C.A., Lasserson D.S., Hobbs F.D. (2016). Global Prevalence of Chronic Kidney Disease—A Systematic Review and Meta-Analysis. PLoS ONE.

[4] van der Plas W.Y., Noltes M.E., van Ginhoven T.M., Kruijff S. (2020). Secondary and Tertiary Hyperparathyroidism: A Narrative Review. Scand. J. Surg..

[5] Levin A., Bakris G.L., Molitch M., Smulders M., Tian J., Williams L.A., Andress D.L. (2007). Prevalence of abnormal serum vitamin D, PTH, calcium, and phosphorus in patients with chronic kidney disease: Results of the study to evaluate early kidney disease. Kidney Int..

[6] Adam M.A., Untch B.R., Danko M.E., Stinnett S., Dixit D., Koh J., Marks J.R., Olson J.A. (2010). Severe obesity is associated with symptomatic presentation, higher parathyroid hormone levels, and increased gland weight in primary hyperparathyroidism. J. Clin. Endocrinol. Metab..

[7] Grey A.B., Evans M.C., Stapleton J.P., Reid I.R. (1994). Body weight and bone mineral density in postmenopausal women with primary hyperparathyroidism. Ann. Intern. Med..

[8] Vijgen G.H., Sparks L.M., Bouvy N.D., Schaart G., Hoeks J., van Marken Lichtenbelt W.D., Schrauwen P. (2013). Increased oxygen consumption in human adipose tissue from the “brown adipose tissue” region. J. Clin. Endocrinol. Metab..

[9] Blondin D.P., Labbé S.M., Tingelstad H.C., Noll C., Kunach M., Phoenix S., Guérin B., Turcotte E.E., Carpentier A.C., Richard D. (2014). Increased brown adipose tissue oxidative capacity in cold-acclimated humans. J. Clin. Endocrinol. Metab..

[10] Niemann B., Haufs-Brusberg S., Puetz L., Feickert M., Jaeckstein M.Y., Hoffmann A., Zurkovic J., Heine M., Trautmann E.M., Müller C.E. (2022). Apoptotic brown adipocytes enhance energy expenditure via extracellular inosine. Nature.

[11] Kajimura S., Spiegelman B.M., Seale P. (2015). Brown and Beige Fat: Physiological Roles beyond Heat Generation. Cell Metab..

[12] Scheele C., Wolfrum C. (2019). Brown Adipose Crosstalk in Tissue Plasticity and Human Metabolism. Endocr. Rev..

[13] Becher T., Palanisamy S., Kramer D.J., Eljalby M., Marx S.J., Wibmer A.G., Butler S.D., Jiang C.S., Vaughan R., Schöder H. (2021). Brown adipose tissue is associated with cardiometabolic health. Nat. Med..

[14] Cannon B., Nedergaard J. (2004). Brown adipose tissue: Function and physiological significance. Physiol. Rev..

[15] Cypess A.M., Doyle A.N., Sass C.A., Huang T.L., Mowschenson P.M., Rosen H.N., Tseng Y.H., Palmer E.L., Kolodny G.M. (2013). Quantification of human and rodent brown adipose tissue function using 99mTc-methoxyisobutylisonitrile SPECT/CT and 18F-FDG PET/CT. J. Nucl. Med..

[16] Klaus S., Ely M., Encke D., Heldmaier G. (1995). Functional assessment of white and brown adipocyte development and energy metabolism in cell culture. Dissociation of terminal differentiation and thermogenesis in brown adipocytes. J. Cell Sci..

[17] Klaus S. (1997). Functional differentiation of white and brown adipocytes. Bioessays.

[18] Madar I., Isoda T., Finley P., Angle J., Wahl R. (2011). 18F-fluorobenzyl triphenyl phosphonium: A noninvasive sensor of brown adipose tissue thermogenesis. J. Nucl. Med..

[19] Sampath S.C., Sampath S.C., Bredella M.A., Cypess A.M., Torriani M. (2016). Imaging of Brown Adipose Tissue: State of the Art. Radiology.

[20] Gifford A., Towse T.F., Walker R.C., Avison M.J., Welch E.B. (2016). Characterizing active and inactive brown adipose tissue in adult humans using PET-CT and MR imaging. Am. J. Physiol. Endocrinol. Metab..

[21] Lee M.W., Lee M., Oh K.J. (2019). Adipose Tissue-Derived Signatures for Obesity and Type 2 Diabetes: Adipokines, Batokines and MicroRNAs. J. Clin. Med..

[22] Poekes L., Lanthier N., Leclercq I.A. (2015). Brown adipose tissue: A potential target in the fight against obesity and the metabolic syndrome. Clin. Sci..

[23] Zeng M., Liu W., Zha X., Tang S., Liu J., Yang G., Mao H., Yu X., Sun B., Zhang B. (2019). 99mTc-MIBI SPECT/CT imaging had high sensitivity in accurate localization of parathyroids before parathyroidectomy for patients with secondary hyperparathyroidism. Ren. Fail..

[24] Duvall W.L., Case J., Lundbye J., Cerqueira M. (2021). Efficiency of tetrofosmin versus sestamibi achieved through shorter injection-to-imaging times: A systematic review of the literature. J. Nucl. Cardiol..

[25] Ovčariček P.P., Giovanella L., Gasset I.C., Hindié E., Huellner M.W., Luster M., Piccardo A., Weber T., Talbot J.N., Verburg F.A. (2021). The EANM practice guidelines for parathyroid imaging. Eur. J. Nucl. Med. Mol. Imaging.

[26] Maublant J.C., Moins N., Gachon P., Renoux M., Zhang Z., Veyre A. (1993). Uptake of technetium-99m-teboroxime in cultured myocardial cells: Comparison with thallium-201 and technetium-99m-sestamibi. J. Nucl. Med..

[27] Arbab A.S., Koizumi K., Toyama K., Araki T. (1996). Uptake of technetium-99m-tetrofosmin, technetium-99m-MIBI and thallium-201 in tumor cell lines. J. Nucl. Med..

[28] de Jong M., Bernard B.F., Breeman W.A., Ensing G., Benjamins H., Bakker W.H., Visser T.J., Krenning E.P. (1996). Comparison of uptake of 99mTc-MIBI, 99mTc-tetrofosmin and 99mTc-Q12 into human breast cancer cell lines. Eur. J. Nucl. Med..

[29] Wackers F.J., Berman D.S., Maddahi J., Watson D.D., Beller G.A., Strauss H.W., Boucher C.A., Picard M., Holman B.L., Fridrich R. (1989). Technetium-99m hexakis 2-methoxyisobutyl isonitrile:human biodistribution, dosimetry, safety, and preliminary comparison to thallium-201 for myocardial perfusion imaging. J. Nucl. Med..

[30] Marshall R.C., Leidholdt E.M., Zhang D.Y., Barnett C.A. (1990). Technetium-99m hexakis 2-methoxy-2isobutyl isonitrile and thallium-201 extraction, washout, and retention at varying coronary flow rates in rabbit heart. Circulation.

[31] Belhocine T., Shastry A., Driedger A., Urbain J.L. (2007). Detection of 99mTc-sestamibi uptake in brown adipose tissue with SPECT-CT. Eur. J. Nucl. Med. Mol. Imaging..

[32] Moon S.H., Lee Y.S., Lee D.S., Chung J.K., Jeong J.M. (2017). A study of 99mTc-sestamibi labeling condition using radio-chromatography. J. Radiopharm. Mol. Probes.

[33] Rosen E.D., Spiegelman B.M. (2014). What we talk about when we talk about fat. Cell.

[34] Ricquier D. (2011). Uncoupling protein 1 of brown adipocytes, the only uncoupler: A historical perspective. Front. Endocrinol..

[35] Cypess A.M., Kahn C.R. (2010). Brown fat as a therapy for obesity and diabetes. Curr. Opin. Endocrinol. Diabetes. Ob..

[36] Bolland M.J., Grey A.B., Gamble G.D., Reid I.R. (2005). Association between primary hyperparathyroidism and increased body weight: A meta-analysis. J. Clin. Endocrinol. Metab..

[37] Cypess A.M., Lehman S., Williams G., Tal I., Rodman D., Goldfine A.B., Kuo F.C., Palmer E.L., Tseng Y.H., Doria A. (2009). Identification and importance of brown adipose tissue in adult humans. N. Engl. J. Med..

[38] van Marken Lichtenbelt W.D., Vanhommerig J.W., Smulders N.M., Drossaerts J.M., Kemerink G.J., Bouvy N.D., Schrauwen P., Teule G.J. (2009). Cold activated brown adipose tissue in healthy men. N. Engl. J. Med..

[39] Saito M., Okamatsu-Ogura Y., Matsushita M., Watanabe K., Yoneshiro T., Nio-Kobayashi J., Iwanaga T., Miyagawa M., Kameya T., Nakada K. (2009). High incidence of metabolically active brown adipose tissue in healthy adult humans: Effects of cold exposure and adiposity. Diabetes..

[40] Admiraal W.M., Holleman F., Bahler L., Soeters M.R., Hoekstra J.B., Verberne H.J. (2013). Combining 123I-metaiodobenzylguanidine SPECT/CT and 18F-FDG PET/CT for the assessment of brown adipose tissue activity in humans during cold exposure. J. Nucl. Med..

[41] Ouellet V., Labbe S.M., Blondin D.P., Phoenix S., Guérin B., Haman F., Turcotte E.E., Richard D., Carpentier A.C. (2012). Brown adipose tissue oxidative metabolism contributes to energy expenditure during acute cold exposure in humans. J. Clin. Investig..

[42] Orava J., Nuutila P., Lidell M.E., Oikonen V., Noponen T., Viljanen T., Scheinin M., Taittonen M., Niemi T., Enerbäck S. (2011). Different metabolic responses of human brown adipose tissue to activation by cold and insulin. Cell Metab..

[43] Hadi M., Chen C.C., Whatley M., Pacak K., Carrasquillo J.A. (2007). Brown fat imaging with (18)F-6-fluorodopamine PET/CT, (18)F-FDG PET/CT, and (123)I-MIBG SPECT: A study of patients being evaluated for pheochromocytoma. J. Nucl. Med..

[44] Izzi-Engbeaya C., Salem V., Atkar R.S., Dhillo W.S. (2014). Insights into Brown Adipose Tissue Physiology as Revealed by Imaging Studies. Adipocyte.

[45] Honek J., Lim S., Fischer C., Iwamoto H., Seki T., Cao Y. (2014). Brown adipose tissue, thermogenesis, angiogenesis: Pathophysiological aspects. Horm. Mol. Biol. Clin. Investig..

[46] Hanssen M.J., Wierts R., Hoeks J., Gemmink A., Brans B., Mottaghy F.M., Schrauwen P., Lichtenbelt W.D.V.M. (2014). Glucose uptake in human brown adipose tissue is impaired upon fastinginduced insulin resistance. Diabetologia.

[47] Santhanam P., Solnes L., Hannukainen J.C., Taïeb D. (2015). Adiposity-related cancer and functional imaging of brown adipose tissue. Endocr. Pract..

[48] Cypess A.M., Weiner L.S., Roberts-Toler C., Franquet Elía E., Kessler S.H., Kahn P.A., English J., Chatman K., Trauger S.A., Doria A. (2015). Activation of human brown adipose tissue by a b3-adrenergic receptor agonist. Cell Metab..

[49] Gnad T., Scheibler S., von Kügelgen I., Scheele C., Kilić A., Glöde A., Hoffmann L.S., Reverte-Salisa L., Horn P., Mutlu S. (2014). Adenosine activates brown adipose tissue and recruits beige adipocytes via A2A receptors. Nature.

[50] Jalloul W., Tibu R., Ionescu T.M., Stolniceanu C.R., Grierosu I., Tarca A., Ionescu L., Ungureanu M.C., Ciobanu D., Ghizdovat V. (2021). Personalized nuclear imaging protocol in cases with nodular goiter and parathyroid adenoma. Acta. Endocrinol..

[51] Eslamy H.K., Ziessman H.A. (2008). Parathyroid scintigraphy in patients with primary hyperparathyroidism:99m Tc Sestamibi SPECT and SPECT/ CT 1. Radiographics.

[52] Petrovic N., Walden T.B., Shabalina I.G., Timmons J.A., Cannon B., Nedergaard J. (2010). Chronic peroxisome proliferator-activated receptor gamma (PPARgamma) activation of epididymally derived white adipocyte cultures reveals a population of thermogenically competent, UCP1-containing adipocytes molecularly distinct from classic brown adipocytes. J. Biol. Chem..

[53] Vitali A., Murano I., Zingaretti M.C., Frontini A., Ricquier D., Cinti S. (2012). The adipose organ of obesity-prone C57BL/6J mice is composed of mixed white and brown adipocytes. J. Lipid Res..

[54] Seale P., Bjork B., Yang W., Kajimura S., Chin S., Kuang S., Scimè A., Devarakonda S., Conroe H.M., Erdjument-Bromage H. (2008). PRDM16 controls a brown fat/skeletal muscle switch. Nature.

[55] Bilezikian J.P., Cusano N.E., Khan A.A., Liu J.M., Marcocci C., Bandeira F. (2016). Primary hyperparathyroidism. Nat. Rev. Dis. Primers..

[56] He Y., Liu R.X., Zhu M.T., Shen W.B., Xie J., Zhang Z.Y., Chen N., Shan C., Guo X.Z., Lu Y.D. (2019). The browning of white adipose tissue and body weight loss in primary hyperparathyroidism. EBioMedicine.

[57] Kamycheva E., Sundsfjord J., Jorde R. (2004). Serum parathyroid hormone level is associated with body mass index. The 5th Tromsø study. Eur. J. Endocrinol..

[58] Di Monaco M., Castiglioni C., Vallero F., Di Monaco R., Tappero R. (2013). Parathyroid hormone is significantly associated with body fat compartment in men but not in women following a hip fracture. Aging Clin. Exp. Res..

[59] Mendoza-Zubieta V., Gonzalez-Villaseñor G.A., Vargas-Ortega G., Gonzalez B., Ramirez-Renteria C., Mercado M., Molina-Ayala M.A., Ferreira-Hermosillo A. (2015). High prevalence of metabolic syndrome in a mestizo group of adult patients with primary hyperparathyroidism (PHPT). BMC Endocr. Disord..

[60] Procopio M., Barale M., Bertaina S., Sigrist S., Mazzetti R., Loiacono M., Mengozzi G., Ghigo E., Maccario M. (2014). Cardiovascular risk and metabolic syndrome in primary hyperparathyroidism and their correlation to different clinical forms. Endocrine.

[61] Tassone F., Gianotti L., Baffoni C., Cesario F., Magro G., Pellegrino M., Emmolo I., Maccario M., Borretta G. (2012). Prevalence and characteristics of metabolic syndrome in primary hyperparathyroidism. J. Endocrinol. Investig..

